# Functional and Genetic Insights into the Role of the *NR4A1* Gene in the Litter Size of the Shaanbei White Cashmere Goat

**DOI:** 10.3390/ani15121729

**Published:** 2025-06-11

**Authors:** Ebadu Areb, Yutian Bi, Yangyang Bai, Qihui Zhu, Lingyuan Ma, Chuanying Pan, Xiaolei Chen, Xianyong Lan

**Affiliations:** 1 Key Laboratory of Animal Genetics, Breeding and Reproduction of Shaanxi Province, College of Animal Science and Technology, Northwest A & F University, Xianyang 712100, China; ebaduareb3@nwafu.edu.cn (E.A.); biyutian0927@163.com (Y.B.); bai345@126.com (Y.B.); zqhzqhzqh2002@163.com (Q.Z.); malingyuan15@163.com (L.M.); panyu1980@126.com (C.P.); 2 College of Science, Northwest A & F University, Xianyang 712100, China; 3 Central Ethiopia Agricultural Research Institute at Worabe Agricultural Research Center, Worabe P.O. Box 021, Ethiopia

**Keywords:** goat, gene expression, genetic variation, litter size, *NR4A1 gene*, association analysis

## Abstract

Nuclear receptor subfamily 4 group A member 1 (*NR4A1*) plays a crucial role in regulating various physiological processes. As gene mining for reproductive traits is essential, this study aimed to investigate the mRNA expression, genetic variation, and association of the *NR4A1* gene with goat litter size. We examined the mRNA expression levels of the *NR4A1* gene in eight different tissues of female Shaanbei White Cashmere (SBWC) goats (*n* = 6). Then, a novel 11-bp insertion/deletion (InDel) variant was genotyped in 1136 SBWC goats, 87 SNPs were identified through resequencing (*n* = 120), and selection signal analysis was undertaken. The *NR4A1* gene was expressed in all examined tissues, including the ovary and the oviduct, suggesting its role in goat reproduction. Both the 11-bp InDel and 13 SNP variants showed significant association with litter size. Additionally, four potential transcription factor binding sites were predicted within the insertion allele, which may contribute to increased litter size. Selection signal analysis revealed strong pressure on the *NR4A1* gene region in the Cashmere goat population. These findings suggest that *NR4A1* is a promising candidate gene for improving litter size in goats and could be utilized as a genetic marker in breeding programs.

## Simple Summary

Nuclear receptor subfamily 4 group A member 1 (*NR4A1*) is a transcription factor that plays a significant role in various physiological activities, including cell proliferation and differentiation, as well as reproductive signaling pathways. In this study, we identified genetic variations in the *NR4A1* gene, both insertion/deletion (InDel) and single nucleotide polymorphism (SNP), that are associated with litter size in goats. These findings offer new insights into the role of *NR4A1* in goat reproduction. Therefore, *NR4A1* could serve as a potential molecular marker for improving reproductive performance in goat breeding programs.

## 1. Introduction

The litter size trait has a significant economic value, which is regulated by genetic and non-genetic factors [1]. Hereditary genetic factors that control litter size include a combination of major genes and polygenes [1]. Several biological processes, such as folliculogenesis and ovulation [2], signal transduction pathways [2], hormonal regulation [3], neuroendocrine regulation [2], and genetic factors, such as the *BMPR1P* gene, play a role in reproductive processes and influence litter size [4]. The low-heritability nature of litter size and maternal effect results in prolificacy traits’ improvement being challenging [5]. To alleviate this challenge, a candidate gene approach in goat genomics is increasingly utilized to uncover the molecular mechanisms underlying variations in traits [6]. The Shaanbei White Cashmere (SBWC) goat is a widely recognized goat breed and valued for its high-quality cashmere and superior meat quality [7] but characterized by low litter size performance [8]. Various studies have reported differing litter size performance values for this breed at different times, including 1.5 [9], 1.7 [10], 1.5 [11], and 1.38 [12]. Therefore, further exploration of candidate genes is required for the breed improvement program.

While increasing litter size has long been a desirable trait in genetic improvement programs for enhanced productivity, this trend raises significant concerns regarding animal welfare and sustainability [13]. Large litters, especially in pigs and small ruminants, are often associated with higher pre-weaning mortality, lower average birth weights, and increased variability among littermates, which can compromise neonatal survival and long-term health [13,14]. Moreover, the metabolic demands placed on dams may elevate risks of exhaustion, insufficient maternal care, and impaired reproductive longevity [13,15]. Therefore, breeding strategies should emphasize optimal litter size rather than maximal, integrating phenotypic traits like birth weight uniformity, maternal behavior, and survival rates to align with animal welfare standards and sustainability [14,16,17].

Nuclear receptor subfamily 4 group A member 1 (*NR4A1*), also referred to as *Nur 77* or *TR3*, belongs to the orphan nuclear receptor transcription factor family. The *Capra hircus NR4A1* gene is localized on chromosome 5 with ten exons. The *NR4A1*-*NR4A3* subfamily comprises immediate early genes that are activated by various stimuli, including peptide hormones, growth factors, cytokines, inflammatory and physiological signals, and cellular stress [18]. It was one of the first genes identified as an early response gene, rapidly activated by nerve growth factor in pheochromocytoma cells [19]. *NR4A1* is involved in regulating metabolism, cardiovascular and neurological function, vascular stability, and immune cell homeostasis during inflammation and cancer [20]. It is expressed in different tissues and plays a role in cell proliferation and apoptosis [21]. Overexpressing *NR4A1* accelerated the development of goat intramuscular preadipocytes through the PI3K/AKT pathway [22]. The *NR4A1* gene is not only associated with reproduction and intramuscular preadipocytes but also used as a marker for feed restriction in dairy goats [23]. On the other hand, the reduced methylation level of the *NR4A1* gene affects gene expression in adult sheep longissimus dorsi muscle [24], and it is a glucose indicator, as its expression was related to the insulin sensitivity of skeletal muscle [25]. The expression of *NR4A1* was down-regulated in diabetic mice [26]. Compared with wild mice, *NR4A1* knockout mice were more likely to be obese [26]. This research output indicates that the *NR4A1* gene might have an association with growth traits.

The *NR4A1* gene was one of the up-regulated genes in the ovaries of Xiang pigs with large litter sizes [27]. It is suggested that this gene might play important roles in promoting litter size by increasing the level of steroid and peptide hormone supply through the ovary and facilitating oocyte ovulation and in vivo fertilization [27]. Additionally, this gene plays a role in regulating progesterone synthesis in goat luteal cells [28], indicating its participation in the reproductive process. Furthermore, the adenosine monophosphate-activated protein kinase/*NR4A1* signaling axis is involved in ovarian function changes in premature ovarian insufficiency rats following umbilical cord mesenchymal stem cell transplantation [29].

Similarly, a significant association between the single nucleotide polymorphism (SNP) exploration of *NR4A1* g.3952A>G and the total number of piglets born and weaned for commercial sows was reported [30]. According to the report, Landrace sows with the AG genotype variant exhibited significantly higher body weight and reproductive performance than the GG genotype. Specifically, they produced 2.98 kg more piglets in total litter weight at birth, 2.41 more piglets were born alive, 2.52 more piglets were weaned alive, and 15.68 kg more in total weaning litter weight was achieved [30]. Also, this gene is involved in the cell cycle and differentiation of granulosa cells, theca cells, and oocytes [31]. Similarly, the *NR4A1* gene regulates the expression of key genes involved in ovarian steroidogenesis and enhances ovarian reserve in aged mice [32]. These studies have implicated that the *NR4A1* gene has a role in the regulation of ovulation and luteal function. However, the function of this gene in goat reproductive performance remains unclear.

It is necessary to use marker-assisted selection (MAS) through the gene mining method in goat genomics to find out how genetic differences are caused at the molecular level, with a focus on traits of interest [6]. The previous reports from our group have identified a variety of potential genetic marker candidate genes that could aid goat breeding programs. Some examples are *PPP6C* [9], *AKAP12* [33], *IGF2BP2* [34], *CLSTN2* [12], *SMAD2* [10], *KMT2A* [35], *GATA4* [36], and *KDM6A* [37].

According to the research findings described above, we hypothesized that genetic variants within the *NR4A1* gene might have an effect on litter size by affecting mRNA expression profiles. Among genetic variants, insertion/deletion (InDel) and SNP offer high accuracy and stability for directly detecting gene polymorphisms [9,38]. Therefore, the aim of this study was to investigate the mRNA expression of the *NR4A1* gene in multiple tissues, as well as the association between genetic variants (InDels and SNPs) and goat litter size. This approach aims to facilitate further gene exploration and provide a potential marker for breeding programs.

## 2. Materials and Methods

### 2.1. Bioinformatics Analysis of Regulation and Conservation of the NR4A1 Gene

The networks of physical interaction, pathways, co-localization, co-expression, and other interactions were examined by using the online bioinformatics tool GeneMANIA (https://genemania.org/ accessed on 20 January 2025), for the *NR4A1* gene with other genes. The conservation analysis of this gene across different taxonomic groups was also examined. To do this, the sequence of the *NR4A1* gene for six species was retrieved from the NCBI; these include *Capra hircus* (NC 030812.1), *Bos taurus* (NC 037332.1), *Ovis aries* (NC 056056.1), *Homo sapiens* (NC 000012.12), *Mus musculus* (NC 000081.7), and *Sus scrofa* (NC 010447.5). MEGA11 software (version 11.0.13) was used to perform different species sequence muscle alignment [9]. The evolutionary tree was constructed using the neighbor-joining method in MEGA11 [39], and similarity analysis was performed using the Clustal Omega Multiple Sequence Alignment (MSA) (Clustal Omega, EMBL-EBI, https://www.ebi.ac.uk/jdispatcher/msa/clustalo accessed on 20 January 2025) bioinformatics tool.

### 2.2. Tissue Sample Collection, Total RNA Extraction, and cDNA Synthesis

Tissue samples were collected from healthy SBWC goats from Yulin, Shaanxi Province, China. A total of eight types of tissues (liver, heart, lung, spleen, oviduct, fat, kidney, and ovary) were collected from the selected firstborn female goats (*n* = 6). The age of the selected animals was between 2 and 3 years and in the estrous cycle stage. All goats were maintained under uniform environmental, management, and feeding conditions. To determine the mRNA expression levels of the *NR4A1* gene, total RNA was extracted from the aforementioned tissues by using TRIzol reagent (TaKaRa Biotech Co., Ltd., Dalian, China). The extracted total RNA was stored at −80 °C following the manufacturer’s protocol. RNA purity and quality were evaluated using a NanoDrop ND-1000 spectrophotometer (Thermo Scientific, Waltham, MA, USA) to ensure the A_260_/A_280_ ratio falls within the acceptable range, which is 1.8 to 2.1 [11]. RNA integrity was assessed via 1% agarose gel electrophoresis [9]. First-strand complementary DNA (cDNA) was synthesized using the Prime-Script^TM^ RT reagent kit (TaKaRa Biotech Co., Ltd.) based on the manufacturer’s protocol. The synthesized cDNA was kept at a temperature of −20 °C [40].

### 2.3. Reverse Transcriptase Quantitative Real-Time PCR (RT-qPCR)

The sequences for the *NR4A1* gene and reference gene (*GAPDH*) primers were designed using NCBI’s primer designing tool (https://blast.ncbi.nlm.nih.gov/Blast.cgi accessed on 12 March 2024). The coding sequence of transcript variant X3 (XM_018047732.1) of the *NR4A1* gene and *Capra hircus GAPDH* as reference gene sequences were used to design primers for RT-qPCR (Table 1). Primer pairs were designed from the exon–exon junction to amplify a specific product without nonspecific amplification or genomic DNA contamination. The primers were produced by Sangon Biotech Ltd., Xi’an, China. The cDNA of the tissues of female goats, including the ovary, spleen, liver, oviduct, heart, kidney, fat, and lung, was used to analyze the mRNA expression. The RT-qPCR mixture (20 µL) comprised 2 µL of cDNA (1:100 dilution), 10 µL of 2× SYBR Premix Ex Taq (Takara Biotech), 7.2 µL of distilled water, and 0.4 µL of each forward and reverse primer. To improve the accuracy, three replications were performed for each sample, and relative gene expression was normalized by the fold change of the 2^−ΔΔCt^ method using the reference gene [9]. The RT-qPCR was conducted in real time using the CFX96 Real-Time PCR Detection System (Bio-Rad, Hercules, CA, USA). The thermal cycling protocol was 40 cycles of pre-cycling at 95 °C for 30 s, denaturation at 95 °C for 10 s, and annealing and extension at 60 °C for 30 s.

### 2.4. Animal Selection, Ear Tissue Sample Collection, and Genomic DNA Extraction

Firstborn litter size data were collected from 1136 randomly selected goats from the SBWC goat in Yulin, Shaanxi Province, China. All sampled goats were raised at the Shaanbei Cashmere Goat Breeding Farm in Hengshan County. The Agricultural Technical Station of Yulin City, Shaanxi Province, provided the recorded data. According to the pedigree, there is no genetic relationship between goat individuals. The selected goats were clustered into two based on their birth type (mother with single birth and mother with multiple births). All goats were maintained under suitable and uniform environmental, feeding, and management conditions [9,41]. The sampled goats were bred through natural mating without controlled reproductive techniques. The does were mated with bucks at a ratio of 1:18–24. Does could kid up to 3 times for 2 years, and kids were grown with their does until a weaning age of 3 months [42].

Ear samples were excised and placed in centrifuge tubes containing 70% alcohol for preservation [9]. Genomic DNA was extracted from the ear tissue using a high-salt extraction method. This is a reliable and cost-effective approach for obtaining DNA [43]. DNA quality and purity were assessed by measuring the A_260_/A_280_ ratio using a NanoDrop™ 1000 Spectrophotometer (Thermo Scientific, Waltham, MA, USA) [11]. Then, the extracted DNA was diluted to a concentration of 20 ng/µL and stored at −20 °C for further use [44].

### 2.5. Insertion–Deletion Identification and Primer Design

InDels for the goat *NR4A1* gene were identified by using bioinformatics tools of Ensembl (https://asia.ensembl.org/index.html accessed on 23 March 2024) and GGVD (Goat VariationDB (GGVD) of the Animal Omics database (omicsDB) [45], both database were accessed on 12 March 2024. Additionally, the GGVD database was used to examine minor allele frequencies (MAF), geographical origin group frequencies, and genetic component group frequencies. InDels with >10 bp and MAF > 0.05 were selected to design primers. Primers were designed for identified variants of InDels for polymorphism detection and genotyping by using the NCBI primer designing tool (https://www.ncbi.nlm.nih.gov/tools/primer-blast/ accessed on 23 March 2024). Then, the primers were synthesized by Sangon Biotech Ltd., Xi’an, China (Table 1).

### 2.6. PCR Amplification, Polymorphism Detection, and Genotyping

Polymorphism was detected for the identified InDels by using the DNA mixed pool technique of 24 to 48 randomly selected individuals [9,46]. For the touchdown PCR program, a reaction for mixed pool PCR with a volume of 13 µL was employed. The setup mixture included 0.3 µL of DNA from each individual DNA sample, 6.5 µL of PCR mix, 5 µL of double-distilled water (ddH_2_O), and 0.5 µL of each forward and reverse primer. To enhance detection efficiency, each primer was replicated three times. The mixed pool PCR products were detected on a 3.5% agarose gel and then stained with ethidium bromide. The presence of two distinct bands or differences in band location across the three replicates for a given primer indicated the availability of polymorphism.

PCR amplification for genotyping used a reaction mixture with a total volume of 13 µL with 5 µL of ddH_2_O, 0.5 µL of each forward and reverse primer, 0.5 µL of DNA, and 6.5 µL of PCR mix. The temperature adjustment was an initial denaturation step at 95 °C for 5 min, followed by 35 cycles of denaturation at 94 °C for 30 s, annealing at 65 °C for 30 s, and extension at 72 °C for 20 s with a final extension at 72 °C for 5 min. Additionally, at the final stage, the temperature was maintained at 12 °C for 20 min to preserve and maintain the integrity of the PCR product. Lastly, the PCR product was detected using 3.5% agarose gel electrophoresis, and the gel was stained with ethidium bromide. Subsequently, from each genotype, two PCR products were randomly selected and sent for Sanger sequencing. Sequence synthesization was performed by Sangon Biotech Ltd., Xi’an, China. Then, sequence alignment was performed by using FinchTV version 1.4.0 and BioXM software version 2.7.1.

### 2.7. SNP Loci Identification for the NR4A1 Gene Through Resequencing

By resequencing the 120 SBWC goats, SNP loci were found from the *NR4A1* gene. First, the original FASTQ file was filtered using fastp v0.20.0, and then the whole genome data were aligned with the Oar_rambouillet_v2.0 reference genome using BWA-MEM v0.7.13-r1126 to obtain a binary alignment bam file [47]. SNPs were detected using GATK v3.6-0-g89b7209, and SNP calling was performed using the “HaplotypeCaller” and “GenotypeGVCFs” modules. Finally, bcftools-1.13 was used to remove sequencing and alignment errors, and the parameters were “QD < 2.0,||QUAL < 30.0,||SOR > 3.0,||FS > 60.0, ||MQ < 40.0,||MQRankSum < −12.5,||ReadPosRankSum < −8.0”, with further filtered SNPs using the parameters “-m2 -m2-I”, MAF ≥ 0.05, and F_MISSING < 0.2.

### 2.8. Population Genetic Parameter Estimation

Nei’s method was used to measure genetic parameters of the populations, including the effective allele number (Ne), polymorphism information content (PIC), heterozygosity (He), and homozygosity (Ho) (http://www.msrcall.com/Gdicall.aspx [11], accessed on 2 January 2025). The polymorphism information content value was classified as PIC < 0.25, 0.25 < PIC < 0.5, and PIC > 0.5 for low, moderate, and high diversity, respectively [11]. Similarly, Hardy–Weinberg equilibrium (HWE), Weir and Cockerham (W&C), and allele frequency were calculated by using the Genepop on the Web online tool.

### 2.9. Analysis of Selection Signal at the Region of the NR4A1 Gene

The *NR4A1* gene’s 0.5 Mb flanking region was examined for the strength of selection signals. The Cashmere goat population was compared with goat ancestral groups, such as Bezoar (BEZ), Southwest Asian goat (SWA), African dairy goat (AFD), Europe (EUR), and East Asian goat (EAS). The statistical methods, such as nucleotide diversity (−log10(Pi)), Tajima’s D, heterozygosity (Z(Hp)), integrated haplotype score (|iHS| > 2.0 ratio), cross-population extended haplotype homozygosity (XPEHH), Z-transformed Fst (Z(Fst)), difference in nucleotide diversity (ln(Pi ratio)), and composite likelihood ratio (CLR), were used to determine the selection sweep. A sliding window of 30 kb of the Animal Omics of the GGVD database was used to retrieve the annotation data.

### 2.10. Identification of Transcription Factor Binding Site

An online bioinformatics transcription factor binding site (TFBS) prediction tool, AImodules (https://www.biozentrum.uni-wuerzburg.de/bioinfo/computing/aimodules, accessed on 29 January 2025), using predefined matrices from the JASPAR database, was used. AIModules recognizes conserved motifs and combinations of motifs (modules), allowing for a number of interesting biological applications, such as the analysis of promoter TFBS [48]. For both insertion and deletion sequences of the flanking region, it was used to predict TFBS for InDel mutation.

### 2.11. Data Analysis

The mRNA relative expression levels for various tissues of the *NR4A1* gene were determined by using the 2^−ΔΔCt^ method [9,11]. Nei’s online tool was used to analyze population genetic parameters, such as Ho, He, Ne, and PIC values. Additionally, the Genepop online tool was used to analyze HWE, allele frequency, and W&C parameters. Similarly, to detect the linkage disequilibrium (LD) between SNPs of the *NR4A1* gene, D’ (normalized measure of linkage) and r^2^ (squared correlation coefficient) were estimated and visualized by using the SHEsis online program (http://analysis.bio-x.cn [49], accessed on 2 January 2025). The linkage disequilibrium threshold was categorized as D’ or r^2^ > 0.8, 0.5 < r^2^ < 0.8, and r^2^ < 0.5, which were high, moderate, and low, respectively [50]. GraphPad Prism version 10.4.1 was used to determine the chi-square test (χ^2^) of the relative mRNA expression profile across tissues and allele and genotype frequency for the sampled population. The association between litter size (single-born mother vs. multiple-born mother) (odds of multiple kids) and the 11-bp InDel genotype (II, ID, and DD) was analyzed by using a binomial logistic regression model in R software version 2024.12.1 [51].

(1) $$\log\left(\frac{P\left(multiple\;kid\right)}{1-P\left(multiple\;kid\right)}\right)=\beta 0+\beta 1\;x\;Genotype\;ID+\beta 2\;x\;Genotype\;II$$

where *P* represents the probability of multiple kids, with the DD genotype used as the reference category. The reference genotype was taken alphabetically by default. On the other hand, SPSS version 20 was used to analyze the association between litter size and SNP variants. The model used was Y_jk_ = µ + G_j_ + e_jk,_ where Y_jk_ = litter size, µ = population mean, G_j_ = effect of genotype (each SNP have either 2 or 3 levels), and e_jk_ = random error.

The gene regulatory network was visualized by using the platform https://genemania.org accessed on 2 January 2025). GeneMania allows for visualization of up to 100 genes linked to a query gene; however, in this study, only 30 genes were selected to construct the network diagram in order to enhance clarity and simplify the visualization. For the graphical network visualization of the *NR4A1* gene, multiple interaction parameters were used, including physical interaction, co-expression, co-localization, genetic interaction, pathway involvement, and shared protein domains. A maximum of 30 resultant genes were allowed for automatically selected weighting method. A network with thicker lines indicates strong evidence of linkage [52]. Selection signal statistical methods, such as -log10(Pi), Tajima’s D, Z(Hp), ln(Pi ratio), |iHS| > 2.0 ratio, XPEHH, Z(Fst), and CLR, were determined by using annotation data of GGVD under the Animal Omics database.

## 3. Results

### 3.1. NR4A1 Gene Interaction Network

**Figure 1:**
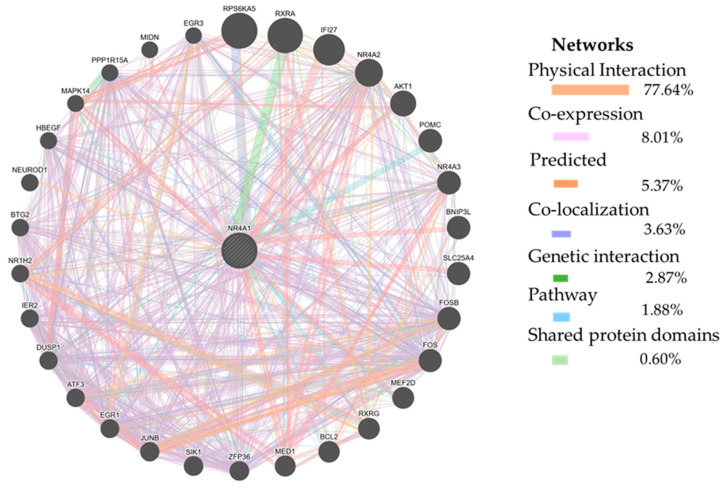
Prediction of regulatory network for *NR4A1* gene. Thirty gene interaction networks, including genes, transcription factors, and pathways that have been directly or indirectly associated with the *NR4A1* gene, were identified. The network’s legend indicates the proportions of physical interaction, co-expression, predicted co-localization, genetic interaction, pathways, and shared protein domains with regulatory networks.

According to GeneMania, (https://genemania.org/ accessed on 2 January 2025), the *NR4A1* gene physically and genetically interacted with, co-expressed, co-localized, and shared protein domains with other genes and transcription factors (Figure 1). Among many genes, only the top 30 genes were filtered out to construct a network diagram. Among the listed genes, the majority of them have a physical interaction with the *NR4A1* gene (77.64%) and are co-expressed (8.01%) (Figure 1). Genes like *NR4A2*, *EGR1*, *ATF3*, *ZFP36*, and others were co-expressed with *NR4A1*. On the other hand, genes like *IFI27*, *AKT1*, *SLC25A4*, *BNIP3L*, and others have a physical interaction with the *NR4A1* gene. Similarly, *RXRA*, *RXRG*, *IFI27*, and others have a direct genetic interaction with the *NR4A1* gene.

### 3.2. NR4A1 Gene Conservation Analysis Across Taxonomic Order

**Figure 2:**
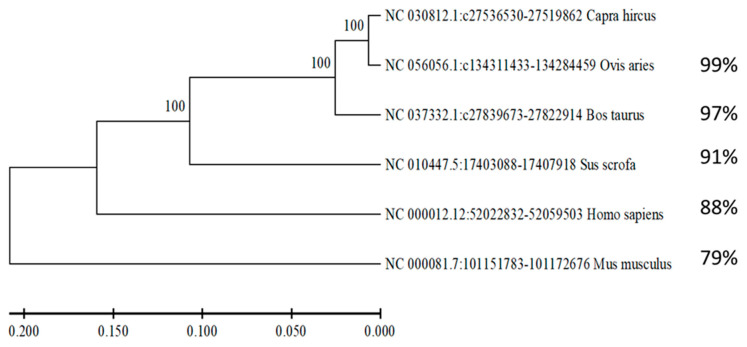
Phylogeny tree for the *NR4A1* gene across different species. The evolutionary conservation of the *NR4A1* gene for *Capra hircus*, *Ovis aries*, *Bos taurus*, *Sus scrofa*, *Homo sapiens*, and *Mus musculus* with their similarity percentage was determined.

Five other species’ nucleotide sequences were compared to the goat *NR4A1* gene. The taxonomic order of the species, including artiodactyl (*Ovis aries*, *Bos taurus*, and *Sus scrofa*), primate (*Homo sapiens*), and rodent (*Mus musculus*), was used for comparison. Artiodactyls, or even-toed ungulates, exhibit a high degree of similarities among taxonomic orders. In particular, the sequences of *Ovis aries* (NC 056056.1), *Bos taurus* (NC 037332.1), *Sus scrofa* (NC 010447.5), *Homo sapiens* (NC 000012.12), and *Mus musculus* (NC 000081.7) were found to have homological similarity with *Capra hircus* of 99%, 97%, 91%, 88%, and 79%, respectively. The resulting phylogenetic tree shows a more distant relationship with primates and rodents but a close evolutionary relationship among goats, sheep, and cattle (Figure 2).

### 3.3. NR4A1 Gene mRNA Transcriptional Profiles

**Figure 3:**
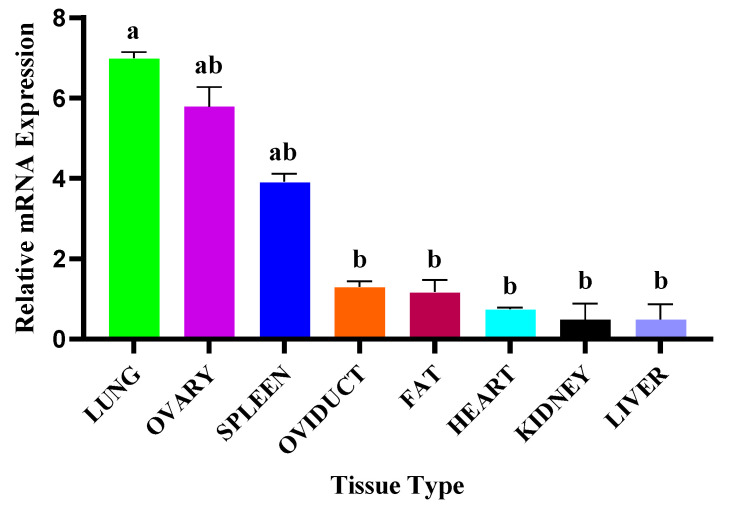
Relative mRNA expression profile of the *NR4A1* gene in tissues of the SBWC goat. Tissues from the lung, ovary, spleen, oviduct, fat, heart, kidney, and liver of six firstborn female goats were evaluated. Data represent means ± SEM; letters on the bar (a, b) represent significant difference of means at *p* < 0.05.

The relative mRNA expression profile of the *NR4A1* gene (NC 030812.1 chromosome 5) across various tissues of the firstborn female SBWC goat was evaluated. For both *NR4A1* and *GAPDH*, a single sharp peak of the melting curve was observed at approximately 87 °C and 83 °C, respectively, confirming the specificity of the amplified product. This gene was expressed in all tissues examined, including the lung, ovary, spleen, oviduct, fat, heart, kidney, and liver. The lung, ovary, and spleen had relatively high expression of the *NR4A1* gene compared to others (*p* < 0.05). Moderate expression in the ovary could suggest a role in reproductive functions (Figure 3). However, minimal expression includes the liver, heart, kidney, and others, indicating that the gene may have less relevance for the metabolic or circular functions.

### 3.4. Genotyping and Population Genetic Parameter Analysis

**Figure 4:**
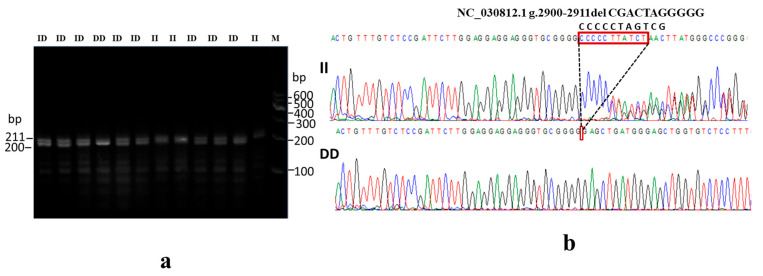
Identification of the 11 bp-del of nucleotide sequence variants within the goat *NR4A1* gene. (**a**) Gel electrophoresis diagram with variant genotypes of II, homozygote insertion/insertion; ID, heterozygote insertion/deletion; DD, homozygote deletion/deletion; M, marker; and bp, base pair. (**b**) Sanger sequencing diagram of PCR product. The upper panel shows the sequence chromatograms of the II genotype, whereas the lower one shows the DD genotype, and the dashed line highlighted triangle indicates the inserted and deleted sequence.

To detect polymorphism, eight primer pairs were designed from both upstream and intron regions of the *NR4A1* gene. After mixed pool detection, a novel variant of primer 3 (NC_030812.1 g.2900-2911del CGACTAGGGGG), exhibited polymorphism (Table 1). Three genotyping variants were identified from 1136 firstborn female goats through PCR amplification, gel electrophoresis (Figure 4a), and Sanger sequencing (Figure 4b). The genotypes include insertion/insertion (II: *n* = 245), insertion/deletion (ID: *n* = 637), and deletion/deletion (DD: *n* = 254) (Table 2). According to the sampled population, the genotype frequency of a population was estimated as 21.6%, 56.1%, and 22.4% for II, ID, and DD, respectively. Additionally, homozygosity and heterozygosity population parameters were nearly equal in proportion with the sampled population (Table 2). The detailed analysis of genetic variation within a population based on genotypes and allele frequencies, as well as metrics that measure genetic diversity within a population, is illustrated in Table 2.

The proportionality of the nearly equal chance of both alleles (I and D) was confirmed by the effective allele number (Ne), which was 1.999 (Table 2). The PIC value (0.374) indicates that the population has a moderate diversity. On the other hand, according to the chi-square test (χ^2^) of the sampled population, the novel variant NC_030812.1 g.2900-2911del CGACTAGGGGG of the *NR4A1* gene in the SBWC goat population significantly deviates from HWE (*p* < 0.05) (Table 2). The HWE deviation was also confirmed by the W&C statistical parameter. The W&C value of −0.1211 indicated that the population at the target locus shows moderate heterozygote excess (12.11%) compared to expectations under HWE. This could suggest heterozygote advantage or gene flow in the population. This might be due to the selection program of SBWC goats for the breed improvement program, including growth and reproduction improvement, which results in non-random mating, and, simultaneously, the genomic region of the *NR4A1* gene might be under selection pressure.

On the other hand, according to the SNP analysis results, the population was in HWE, except for SNP 8. The genetic parameters, such as Ho, He, Ne, and PIC, for each SNP were different. Similarly, there is moderate diversity for the population because the PIC values for SNP2 and SNP8 were 0.27 (Table 3). According to the Ne value, all of the SNPs had different allele proportionality. This indicates that the population’s diversity is decreasing due to the selective breeding program for the target goat population. In addition, the values for most of the SNPs, such as SNP1, SNP4, SNP5, SNP6, and others, verified that the population’s genetic diversity is low (Table 3).

Additionally, for both the ancestral and worldwide goat groups, the aforementioned InDel loci had different alternate allele frequencies (Table 4 and Table S1 and supplementary file Figures S1 and S2). Data for six ancestral goat groups and worldwide goats, including both reference and alternate allele frequencies, were obtained from the Animal Omics database. The alternate allele frequency is an important parameter to study variability at a given locus. Populations with higher alternate allele frequency tend to exhibit greater genetic diversity, which can enhance their adaptability and resilience.

### 3.5. Association of NR4A1 Gene InDel Variants with Goat Litter Size

The main effect of the 11bp-del mutation of the *NR4A1* gene significantly impacted litter size (*p* = 1.62 × 10^−9^) (Table 5). Firstborn mothers with the II genotype exhibited approximately four times higher odds of producing multiple kids compared to those with the DD genotype of 11-bp deletion (odds ratio = 3.975, *p* = 1.58 × 10^−6^) (Table 5). Consequently, firstborn SBWC goats carrying the DD genotype for the aforementioned InDel loci had a significantly lower probability of producing multiple kids.

**Figure 5:**
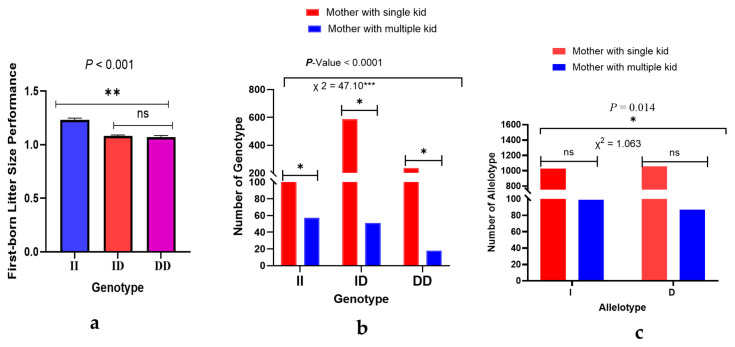
Litter size performance and distribution of the genotypes of the 11-bp-del loci in the first-born mother with single and multiple kid groups of the *NR4A1* gene. (**a**) Litter size performance across different genotypes of firstborn mothers in SBWC goats; data represent the mean ± SEM, and mean values are considered different: ** = *p* < 0.001. (**b**) The intra-group of genotype χ^2^ tests of the 11-bp loci within the *NR4A1* gene in SBWC goats; number of genotype distributions considered different: *** = *p* < 0.0001. (**c**) The intra-group allele type χ^2^ test of the 11-bp locus within the *NR4A1* gene in SBWC goats; data represent the number of allele type distributions for the sampled population. Distribution of alleles considered different: * = *p* < 0.05, ns-not significant.

Mothers with II genotypes have significantly higher litter size performance than ID and DD genotypes (Figure 5a). However, there is no statistically significant difference between mothers with ID and DD genotypes. The distribution of 11 bp-del InDel variations varied among different litter size types according to the χ^2^ test of the entire sampled population of the intra-group result (*p* < 0.0001) (Figure 5b). On the other hand, the χ^2^ test distribution analysis of the mother with single kids and mother with multiple kid groups of SBWC goats showed no statistically significant difference between allotypes at the 11 bp-del loci of the *NR4A1* gene (Figure 5c).

### 3.6. Association of the NR4A1 Gene SNP Loci with Goat Litter Size

SNPs were identified by resequencing the sampled goat population (*n* = 120) for the *NR4A1* gene. The resequencing approach helps to genotype individuals, determine genetic variation, and perform association analysis. A total of 87 SNPs were identified, and 49 SNPs were evaluated for the association analysis. The remaining SNPs did not have enough variability within the sampled population. Among the analyzed SNPs, 13 SNPs were statistically significant with goat litter size (Table 6). SNPs with non-significant associations are available in the supplementary file, Table S2. Among SNPs, SNP1, SNP4, SNP5, SNP6, SNP7, SNP11, and SNP12 were highly significantly associated with litter size (*p* < 0.0001) (Table 6). Certain genotypes within the SNPs, such as TC (SNP1), CC (SNP2), GG (SNP3), CT (SNP5), AG (SNP6), CT (SNP7), CC (SNP9), CC (SNP10), and AA (SNP11), are represented by a low number of individuals (Table 6). This indicates that these variants are rare in the studied samples of individuals. Due to the limited number of individuals with these genotypes, further validation with a larger sample size may be necessary.

### 3.7. Linkage Disequilibrium Estimation Among SNPs

**Figure 6:**
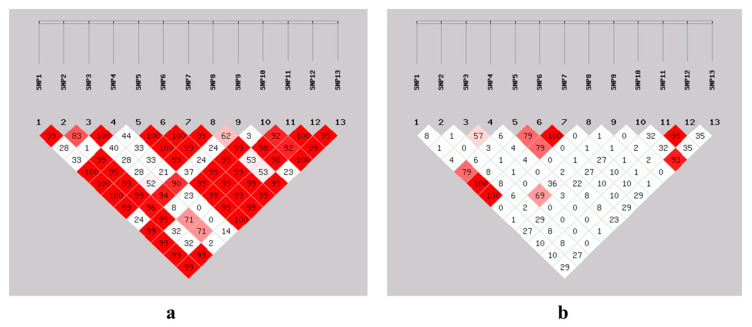
Linkage disequilibrium (LD) analysis between 13 SNPs of the *NR4A1* gene. (**a**) D’ test LD (**b**) r^2^ correlation among SNPs. For both plots, red boxes indicate strong LD, light red boxes indicate moderate LD, and white boxes indicate low or no LD among SNPs.

To determine linkage disequilibrium among 13 SNPs of the *NR4A1* gene, D’ and r^2^ were estimated for SBWC goats. For both graphs, red boxes represent strong linkage disequilibrium, light red represents moderate LD, and white boxes represent weak LD (Figure 6). Most of the SNPs had strong linkage disequilibrium (D’). For example, SNP 1 was highly linked (complete LD, D’ = 1) with SNP 5, SNP 6, and SNP 7, which means they could have been inherited together (Figure 6a). On the other hand, the linkage analysis (correlation coefficient) expressed by r^2^ varied across SNPs (from strong r^2^ = 1 to no association r^2^ = 0) (Figure 6b).

### 3.8. Analysis of Selection Signals on the NR4A1 Gene’s Genomic Region

**Figure 7:**
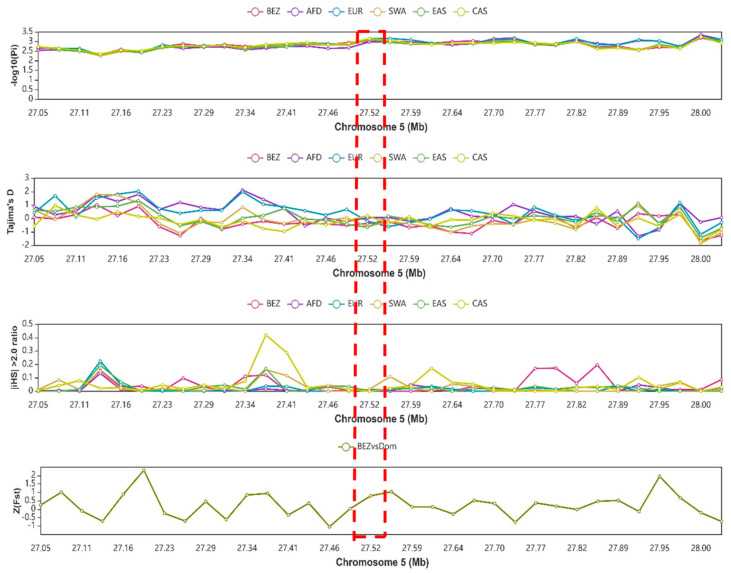
Selective sweep analysis across different statistical methods for the 0.5 Mb flanking region of the *NR4A1* gene: nucleotide diversity (−log10(Pi)); Tajima’s D, iHS (>2.0 ratio), and Z-transformed Fst. The red dashed box refers to the genomic location of the *NR4A1* gene. Ancestral goat groups: Bez = Bezoar, AFD = Africa dairy, EUR = Europe, SWA = Southwest Asia, EAS = East Asia, CAS = Cashmere, and Dom = domestic goat. Mb = megabyte.

When compared to the ancestral group, the *NR4A1* gene’s 0.5 Mb flanking region (shown by a red dashed box) showed stronger selection signals, indicating a higher level of selection pressure (Figure 7). The statistical methods used resulted in observations in Cashmere goats that the ancestral group along chromosome 5 ranged from 27.05 Mb to 28.0 Mb. A positive and peak value of the −log10(Pi) at the genomic region of 27.52 Mb indicated that the region was under selection pressure (Figure 7). Similarly, Tajima’s D and the |iHS| > 2.0 ratio confirmed that the region was under selection. On the other hand, Z (Fst) evidenced that there is a differentiation of Bezoar with domestic goats. Furthermore, other statistics, such as Z(Hp), XPEHH, ln(Pi ratio), and CLR, were used to determine selection sweep and confirm selection pressure of the specified region (Figure S3).

### 3.9. Prediction of Transcription Factor Binding Site

**Figure 8:**
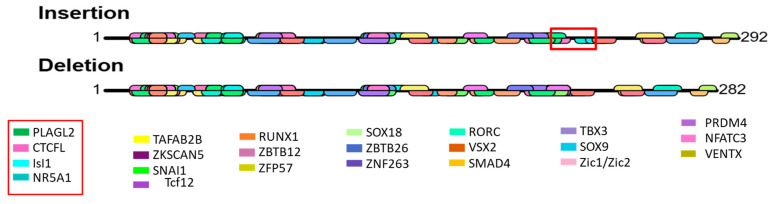
Prediction of transcription factor binding sites for insertion and deletion variants of 11 bp in the goat *NR4A1* gene by using the AImodules online bioinformatics tool. The black line represents the insertion and deletion sequence for the flanking region of InDel. Above the line is the transcription factor binding site for the forward strand (+), and below the line is the transcription factor binding site for the reverse strand (−). Red rectangles highlight binding sites that were found only from insertion sequences.

The distribution of predicted transcription factor binding sites was determined by using insertion and deletion sequences (Figure 8). Many transcriptions factor binding sites were identified by using the AImodule of the online bioinformatics tool. A straight black line indicates the sequence of a gene. Among the detected transcription factor binding sites highlighted in red rectangles (on the graph and the legend), PLAG2 like zinc finger 2 (PLAGL2) (GGGCCCCTA), CCTC-binding factor-like (CTCFL) (GGGCCCCCTAGT), insulin gene enhancer protein 1 (IsI1) (AGCTGATGGCC), and nuclear receptor subfamily 5 group A member 1 (NR5A1) (ATGGCCCTGGT) were only found in the insertion sequence (Figure 8). These transcription factor binding sites (motifs) might have a role in litter size because II genotypes exhibit significantly higher litter size than the other genotypes (Table 5 and Figure 5a).

## 4. Discussion

### 4.1. Function, Evolutionary Conservation, and Expression of Goat NR4A1

In goats, litter size is governed by numerous polygenes with minor effects. Among those polygenes, *NR4A1* was identified as a candidate gene that affects the litter size performance of goats. This gene has been directly or indirectly associated with many other genes (Figure 1). Some of these linked genes directly participate in the reproductive process. For example, *RXRA* and *RXRG* mRNA expression was significantly higher in the endometrial tissue of normal cows compared to repeat breeder cows and repeat breeder cows with subclinical endometritis [53]. The authors explained that altered expression of *RXRA, RXRG*, and related genes could potentially impair embryo elongation and implantation, promoting embryonic loss in repeat breeder cows [53]. Similarly, *EGR1*-ablated (knockout) mice are infertile, highlighting its essential role in reproduction [54]. The study provided mechanistic insights by demonstrating that *ERG1* mediates VEGF A and FDF 2 signaling in buffalo luteal cells, which affects critical reproductive processes, including angiogenesis, cell proliferation, and steroidogenesis in the corpus luteum [54]. Furthermore, genes like *AKT*, which is responsible for placental development and embryo development [55], *NR4A2*, and *NR4A3* are involved in immune response and inflammation, which are essential for successful reproduction [56]. The crucial significance of the *NR4A1* gene was demonstrated by the conservation study, which revealed that it was evolutionarily conserved in *Capra hircus*, *Ovis aries*, *Bos taurus*, *Sus scrofa*, *Homo sapiens*, and *Mus musculus* (Figure 2).

*NR4A1* gene expression was evaluated through RT-qPCR in several tissues. The lung had a comparatively high level of *NR4A1* mRNA expression, followed by the ovary and the spleen. The findings suggest that *NR4A1* may play a significant role in mammalian reproduction, specifically in the maturation of oocytes and follicles. As previously explained, this gene is involved in the cell cycle and differentiation of granulosa cells, theca cells, and oocytes [31]. Moreover, lipid droplets linked to the *NR4A1* contribute to the regulation of progesterone synthesis in the luteal cells of goats [28]. Similarly, *NR4A1–AKR1C1* interaction mediated miR-99b, leading to sustainable progesterone production by the luteal tissue of pigs [57]. It has been shown to regulate the expression of key genes involved in ovarian steroidogenesis and enhance ovarian reserve in aged mouse ovaries [32]. Similarly, several outputs of previous studies have shown that gene expression levels and genetic variations, including InDels and SNPs, significantly influence litter size in goats. For example, studies on genes like *SNX29* and *AKAP12* have identified specific InDel variants strongly associated with increased litter sizes in goat populations. Hence, based on expression results and previously reported evidence, we speculated that the *NR4A1* gene may influence the litter size of goats. To confirm our speculation, the InDel experiment was performed.

### 4.2. Effect of NR4A1 Gene Genetic Variability and Selection Signal

The SBWC goat breed was generally categorized as a low prolific performance breed [8]. Heritability of goat litter size was reported as 0.09 ± 0.02 [58], indicating low heritability and difficulty to improve through direct selection. Therefore, exploration of gene mining for marker-assisted selection is required. As a result, a total of 1136 firstborn does of SBWC goat genomic DNA were utilized to examine the effect of the 11-bp InDel variant in the *NR4A1* gene on goat litter size. According to the association study, goats with the II genotypes had more multiple kids than the ID and DD genotypes (Table 5). Similarly, out of 49 identified SNPs, 13 showed a significant association with litter size. In agreement with the current findings, SNP exploration of *NR4A1* g.3952A>G showed a significant association with litter size at birth and litter size at weaning for commercial sows [30]. Structural variations in a gene can affect its function by disrupting the coding sequence or modifying regulatory regions, leading to changes in protein expression, structure, or activity. [59]. There are various genes that were identified as molecular markers through gene mining for litter size. For example, *DNAH1* has shown increased expression levels in goats with higher litter sizes, emphasizing the necessity of precise genetic research in increasing reproductive performance [60]. Various additional genes were excavated through gene mining techniques to obtain candidate genes for goat litter size. These include *BMP15* and *GDF9* [61], *GNRHR* [62], *SMAD1* [11], *PPP3CA* [63], and *Runx2* [64].

Also, the analysis results of SNPs indicated that most of the SNPs had strong linkage disequilibrium and weak correlations (nearly independent) (Figure 6a,b). These SNPs’ variability may have an effect on individual litter size performance. Through linkage disequilibrium, variation in a single gene can affect phenotypic performance [65]. In agreement with the current findings, two SNPs (P27R and A85G loci) for the *GDF9* gene of SBWC goats were strongly linked [66].

Furthermore, all of the analyzed selective signals suggested that the 11-bp InDel was linked to reproductive traits, as the *NR4A1* gene was under higher selection than in ancestral goat groups (Figure 7). The reason for selecting the Cashmere goat for comparison was due to its genetic relation to the SBWC goat. The SBWC goat is a crossbred breed developed using the Liaoning Cashmere goat as the male line and the Shaanbei Black goat as the female line [67]. Its development involved a straightforward hybridization process and progressed through three key stages: hybrid improvement, crossbreeding stabilization, and selection improvement to upgrade [67]. Also, the reason for selecting Bezoar and other ancestral goat groups was because of their higher alternative allele frequency than others (Table 4 and Supplementary files Figures S1 and S2).

Our investigation used a prediction technique to find transcription factor binding sites because transcription factors can attach to particular DNA sequences. Among the identified transcription factor binding sites, PLAGL2, CTCFL, IsI1, and NR5A1 are capable of binding the transcription factors for the insertion sequence, which results in overexpression of the *NR4A1* gene and has an effect on litter size. Gene regulatory activity is significantly impacted by the orientation and arrangement of transcription factor binding sites [68]. Gene transcription is precisely and specifically regulated by the binding of transcription factors, which also affects the enrollment of the transcriptional mechanism [69]. Our results highlighted the potential relevance of the 11-bp-del and SNP loci of the *NR4A1* gene as a possible marker for marker-assisted selection in goats. In the future, more investigation is needed to uncover the complex molecular pathways that underlie the *NR4A1* gene’s impact on goat reproductive traits.

## 5. Conclusions

This study suggests that the *NR4A1* gene plays a role in goat reproductive performance, particularly litter size. Bioinformatics analysis revealed that *NR4A1* may interact functionally and physically with other reproductive genes, such as *RXRA*, *RXRG*, and *EGR1*. Relative mRNA expression profiling in multiple tissues, including the ovary and the oviduct, supports its potential involvement in fertility-related traits. The association analysis using InDel and SNP markers further indicated significant relationships between *NR4A1* variants and litter size, highlighting its possible utility as a candidate gene for marker-assisted selection in goats. However, the sample size for some variants of SNPs may still be insufficient for a robust conclusion regarding genotypes with low frequency. Therefore, further investigation in a large population is required to ensure consistency. Generally, *NR4A1* appears to be a promising candidate gene for goat litter size, but further research is essential to verify its biological function and potential as a marker in breeding programs.

## Figures and Tables

**Table 1:** Primer pair for polymorphism detection, genotyping, and determining mRNA expression of the *NR4A1* gene.

Primers Name	Primer Sequence (5′->3′)	Sizes (bp)	Tm (°C)	Region
P1	F: TCTGCCTTTGGGACAGCAAG	235	60.54	upstream
	R: GCCTTGAGCCCTATTCACCC		60.47	
P2	F: GGGTGAGATGTGGAGAGCAG	199	59.82	upstream
	R: TAGGGGGATTTGCTCTGTGG		59.08	
P3	F: AGCCCCATCTCTGGACATACT	200	60.06	intron
	R: AATGGGAGCGTTGTCTGGG		60	
P4	F: CACCAGGAAGAGGTCCCAAC	368	59.96	intron
	R: GGGTCAACAGGAGAGGAGGA		60.25	
P5	F: CCTCGCCACACCTTGCATTT	233	61.53	upstream
	R: CACATTCCTCTCCCCACCTC		59.46	
P6	F: AGGCTGTGTGTTTGTCCCAG	197	60.47	intron
	R: GGGTTCGGCCATATCCTCAG		59.96	
P7	F: GGTTGTAAGAAGGCGCAGAG	486	58.92	upstream
	R: GCCCTTCCAACCAATAGCAC		59.18	
P8	F: ACACCTTTACCCGAGAGAGC	223	59.1	intron
	R: ACTCTCTGGACCCTGAACAC		58.66	
*NR4A1*-qPCR	F: ACAGACAGCCTGAAAGGACG	93	59.97	exon 1–2
	R: GACCAGGGAGGTGAGTAGGT		59.96	
*GAPDH*-qPCR	F: AAAGTGGACATCGTTGCCAT	116	58.09	exon 2
	R: CCGTTCTCTGCCTTGACTGT		59.97	

Abbreviations: P = primer; Tm = melting temperature; F = forward; R = reverse; bp = base pair.

**Table 2:** Genetic parameters of the 11 bp InDel locus mutation within the *NR4A1* gene in the SBWC goat.

Genotype Frequencies			Allele Frequencies		Genetic Variation Metrics					
II	ID	DD	I	D	Ho	He	Ne	PIC	HWE	W&C
0.216	0.561	0.224	0.496	0.504	0.500	0.499	1.999	0.374	*p* < 0.05	−0.1211
*n* = 245	*n* =637	*n* = 254								
*N* = 1136										

Where: II = insertion/insertion; ID = insertion/deletion; DD = deletion/deletion; I = insertion; D = deletion; Ho = homozygosity; He = heterozygosity; Ne = effective allele number; PIC = polymorphic information content; HWE = Hardy–Weinberg equilibrium; W&C-Weir and Cockerham.

**Table 3:** Genetic parameters of resequencing locus SNPs within the *NR4A1* gene in the SBWC goat.

SNPs	Genotypes	Genomic Location	Variant ID	Allele Frequencies	Ho	He	Ne	PIC	HWE
SNP1	TT (115)	NC 030812.1: g. 27520435T>C	Novel	0.96 (T)	0.96	0.04	1.04	0.03	*p* > 0.05
	TC (5)			0.04 (C)					
SNP2	GG (76)	NC 030812.1: g. 27521198G>C	rs668853994	0.80 (G)	0.68	0.32	1.48	0.27	*p* > 0.05
	GC (39)			0.20 (C)					
	CC (5)								
SNP3	CC (101)	NC 030812.1: g. 27521415C>G	rs657635772	0.92 (C)	0.85	0.15	1.18	0.14	*p* > 0.05
	CG (18)								
	GG (1)			0.08 (G)					
SNP4	CC (108)	NC 030812.1: g. 27522352C>T	Novel	0.95 (C)	0.91	0.09	1.10	0.09	*p* > 0.05
	CT (12)			0.05 (T)					
SNP5	CC (116)	NC 030812.1: g. 27526206C>T	Novel	0.98 (C)	0.97	0.03	1.03	0.03	*p* > 0.05
	CT (4)			0.02 (T)					
SNP6	AA (115)	NC 030812.1: g. 27527050A>G	rs683100351	0.98 (A)	0.96	0.04	1.04	0.04	*p* > 0.05
	AG (5)			0.02 (G)					
SNP7	CC (115)	NC 030812.1: g. 27527625C>T	rs677241829	0.98 (C)	0.96	0.04	1.04	0.04	*p* > 0.05
	CT (5)			0.02 (T)					
SNP8	CC (81)	NC 030812.1: g. 27528181C>A	Novel	0.79 (C)	0.68	0.32	1.47	0.27	*p* < 0.05
	CA (27)			0.21 (A)					
	AA (10)								
SNP9	GG (96)	NC 030812.1: g. 27531784G>C	rs661365536	0.90 (G)	0.81	0.19	1.23	0.17	*p* > 0.05
	GC (23)			0.10 (C)					
	CC (1)								
SNP10	GG (103)	NC 030812.1: g. 27532725G>A	Novel	0.93 (G)	0.87	0.13	1.15	0.12	*p* > 0.05
	GA (17)			0.07 (A)					
SNP11	CC (3)	NC 030812.1: g. 27533799C>G	rs669578807	0.17 (C)	0.72	0.27	1.38	0.24	*p* > 0.05
	CG (34)			0.83 (G)					
	GG (83)								
SNP12	AA (3)	NC 030812.1: g. 27533803A>G	rs658171703	0.17 (A)	0.72	0.27	1.38	0.24	*p* > 0.05
	AG (34)								
	GG (83)			0.83 (G)					
SNP13	GG (104)	NC 030812.1: g. 27534820G>A	Novel	0.93 (G)	0.88	0.12	1.14	0.12	*p* > 0.05
	GA (16)			0.07 (A)					

SNP = single nucleotide polymorphism; ID = identification; Ho = homozygosity; He = heterozygosity; Ne = effective allele number; PIC = polymorphic information content; HWE = Hardy–Weinberg equilibrium; numbers in the bracket under the genotype column indicate the number of goats; g. = genomic.

**Table 4:** Allele frequency distribution of six ancestral goat groups.

Ancestral Goat Group	Number of Samples	Reference Allele Frequency	Alternate Allele Frequency
Bezoar	24	0.771	0.229
Africa	54	0.713	0.287
Africa Dairy	15	0.767	0.233
Europe	28	0.786	0.214
Southwest Asia	34	0.735	0.265
East Asia	57	0.868	0.132

**Table 5:** Coefficient table of association analysis of 11-bp-del loci in the *NR4A1* gene with litter size (multiple kids born) in SBWC goats.

Coefficient Table						Goodness of Fit		
Predictor	Estimate (β)	SE	z-Value	*p*-Value	Odd Ratio	χ^2^ Test	AIC	*p*-Value
Intercept	−2.5735	0.2445	−10.524	<2 × 10^−16^	0.076	40.484	751.14	1.62 × 10^−9^
Genotype ID	+0.1320	0.2848	+0.463	0.643	1.141			
Genotype II	+1.3801	0.2875	+4.800	1.58 × 10^−6^	3.975			

SE = standard error; AIC = Akaike information criteria.

**Table 6:** Significantly associated resequencing SNPs of the *NR4A1* gene with goat litter size.

SNP	Genotypes	N	Mean ± SEM	*p*-Value
SNP1	TT	115	1.65 ^a^ ± 0.07	8.24 × 10^−17^
	TC	5	1.00 ^b^ ± 0.00	
SNP2	GG	76	1.75 ^a^ ± 0.08	0.032
	GC	39	1.38 ^c^ ± 0.11	
	CC	5	1.60 ^b^ ± 0.24	
SNP3	CC	101	1.70 ^a^ ± 0.07	0.003
	CG	18	1.22 ^b^ ± 0.13	
	GG	1	1.00	
SNP4	CC	108	1.69 ^a^ ± 0.07	5 × 10^−6^
	CT	12	1.08 ^b^ ± 0.08	
SNP5	CC	116	1.65 ^a^ ± 0.07	9.20 × 10^−17^
	CT	4	1.00 ^b^ ± 0.00	
SNP6	AA	115	1.65 ^a^ ± 0.07	8.24 × 10^−17^
	AG	5	1.00 ^b^ ± 0.00	
SNP7	CC	115	1.65 ^a^ ± 0.07	8.24 × 10^−17^
	CT	5	1.00 ^b^ ± 0.00	
SNP8	CC	81	1.52 ^c^ ± 0.08	0.034
	CA	27	1.70 ^b^ ± 0.12	
	AA	10	2.10 ^a^ ± 0.23	
SNP9	GG	96	1.70 ^a^ ± 0.07	0.033
	GC	23	1.35 ^b^ ± 0.13	
	CC	1	1.00	
SNP10	GG	103	1.69 ^a^ ± 0.07	0.001
	GA	17	1.24 ^b^ ± 0.11	
SNP11	CC	3	1.00 ^c^ ± 0.00	1.95 × 10^−11^
	CG	34	1.56 ^b^ ± 0.10	
	GG	83	1.67 ^a^ ± 0.08	
SNP12	AA	3	1.00 ^c^ ± 0.00	1.95 × 10^−11^
	AG	34	1.56 ^b^ ± 0.10	
	GG	83	1.67 ^a^ ± 0.08	
SNP13	GG	104	1.68 ^a^ ± 0.07	0.003
	GA	16	1.25 ^b^ ± 0.11	

SNP = single nucleotide polymorphism; N = number of genotyped animals; letters across columns indicate significant differences within SNPs.

## Data Availability

The raw data supporting the conclusions of this article will be made available by the authors upon request.

## Supplementary Materials

The following supporting information can be downloaded at: https://www.mdpi.com/article/10.3390/ani15121729/s1, Figure S1. Allele frequencies distribution of 11 bp-del locus of *NR4A1* gene for eight ancestral goat groups. The alternate allele frequencies of Africa, Southwest Asia, Africa dairy, and Bezoar ancestral goat groups were relatively higher, which indicates differentiation of the breed through time and results in diversity. Frequencies data was retrieved from the Animal Omics database. Ref = reference allele, Alt = alternate allele; Figure S2. Allele frequencies distribution of 11 bp-del locus of *NR4A1* gene for world-wide goat groups. Relatively higher alternate allele frequencies were available for Morocco, Tanzania, Cashmere, Switzerland, and Saanen goat. These high frequencies indicate that there is higher diversity, which enables adaptation and resilience. Data was generated from the Animal Omics data base. Ref = reference allele, Alt = alternate allele; Figure S3. Selective sweep analysis across different statistical methods for the 0.5 Mb flanking region of the *NR4A1* gene: heterozygosity (Z(Hp)), composite likelihood ratio (CLR), cross-population extended haplotype homozygosity (XPEHH), and difference in nucleotide diversity (ln(Pi ratio)). The red dashed box referred to the genomic location of the *NR4A1* gene. Ancestral goat groups Bez = Bezoar, AFD = Africa dairy, EUR = Europe, SWA = Southwest Asia, EAS = East Asia, CAS = Cashmere, Dom = Domestic goat. Mb = megabyte; Table S1. Allele frequencies distribution of worldwide goat groups for the 11 bp-del locus of the *NR4A1 gene*; Table S2. Selected non-significantly associated resequencing loci SNPs of the *NR4A1* gene with goat litter size.

## Abbreviations

The following abbreviations are used in this manuscript:

SBWC	Shaanbei White Cashmere goat
*NR4A1*	Nuclear receptor subfamily 4 group A member 1
InDel	Insertion–deletion
SNP	Single nucleotide polymorphism
MAS	Marker-assisted selection
MSA	Multiple Sequence Alignment
GGVD	Goat Genomic Variation Database
MAF	Minor allele frequencies
Ne	Effective allele number
PIC	Polymorphism information content
He	Heterozygosity
Ho	Homozygosity
HWE	Hardy–Weinberg equilibrium
W&C	Weir and Cockerham
BEZ	Bezoar goat
SWA	Southwest Asian goat
AFD	African dairy goat
EUR	European goat
EAS	East Asian goat
iHS	Integrated haplotype score
XPEHH	Cross-population extended haplotype homozygosity
Fst	Z-transformed
CLR	Composite likelihood ratio
TFBS	Transcription factor binding site
LD	Linkage disequilibrium
AIC	Akakie Information Criteria
