# Supplementary material for: Functional and Genetic Insights into the Role of the NR4A1 Gene in the Litter Size of the Shaanbei White Cashmere Goat
Source: Animals (Basel). 2025 Jun 11;15(12):1729. doi: 10.3390/ani15121729 (PMC12189067; doi:10.3390/ani15121729)
Supplement: Supplementary file 1 [file animals-15-01729-s001.zip › Suplmentary File Figure.pdf]

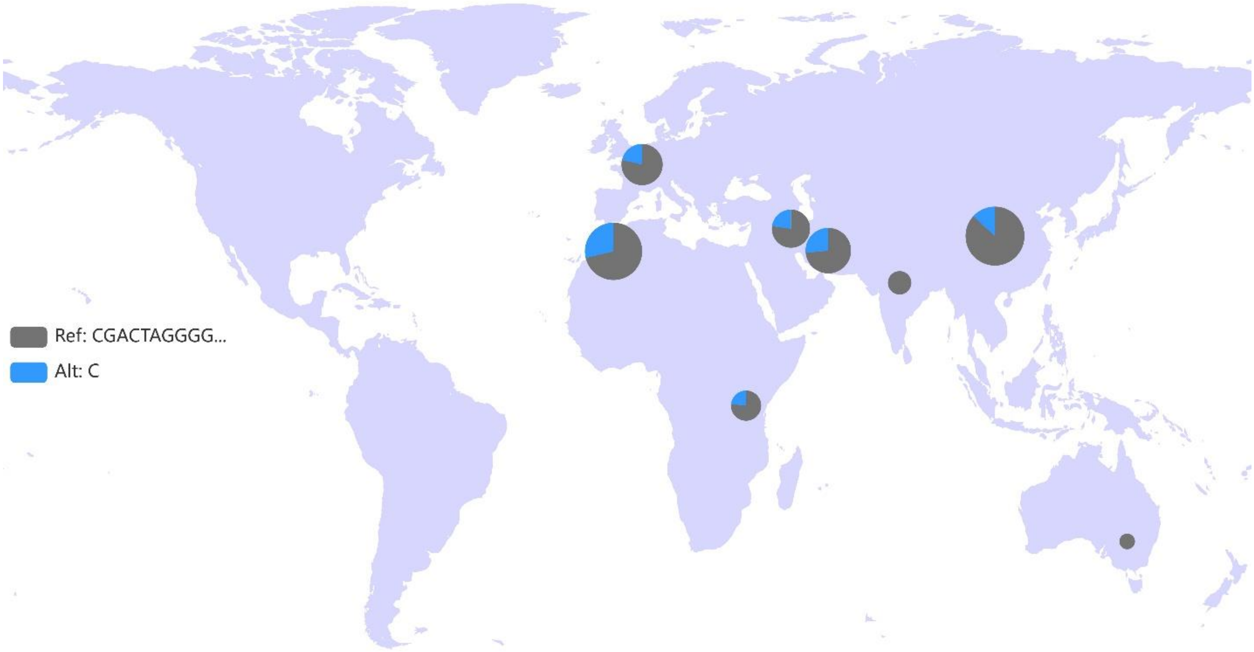

Figure S1. Allele frequencies distribution of 11 bp-del locus of *NR4A1* gene for eight ancestral goat groups. The alternate allele frequencies of Africa, Southwest Asia, Africa dairy, and Bezoar ancestral goat groups were relatively higher, which indicates differentiation of the breed through time and results in diversity. Frequencies data was retrieved from the Animal Omics database. Ref = reference allele, Alt = alternate allele

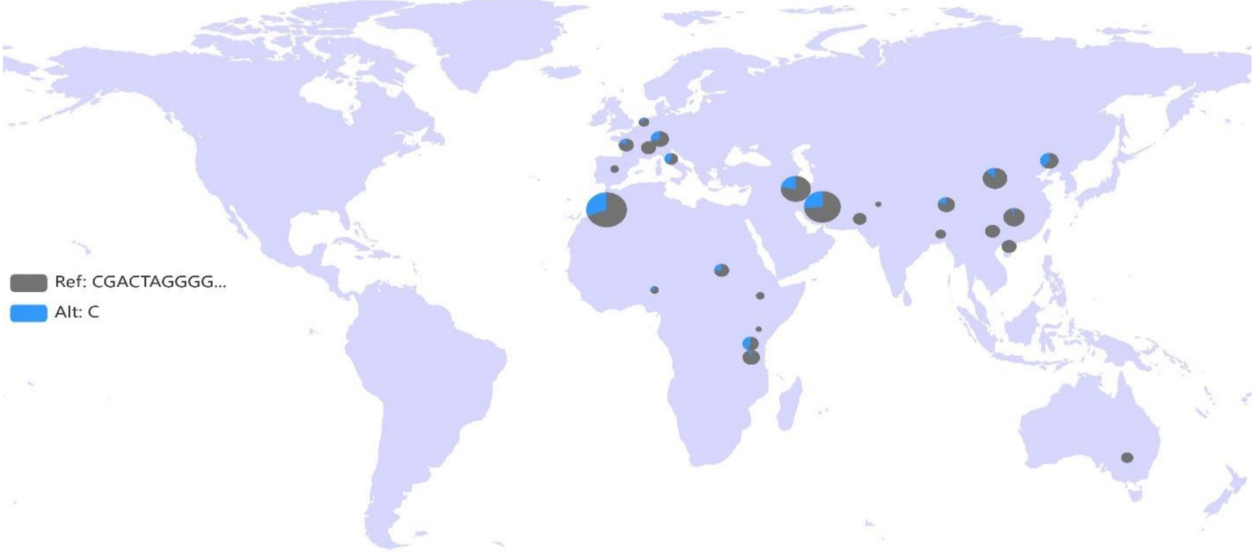

Figure S2. Allele frequencies distribution of 11 bp-del locus of *NR4A1* gene for world-wide goat groups. Relatively higher alternate allele frequencies were available for Morocco, Tanzania, Cashmere, Switzerland, and Saanen goat. These high frequencies indicate that there is higher diversity, which enables adaptation and resilience. Data was generated from the Animal Omics data base. Ref = reference allele, Alt = alternate allele

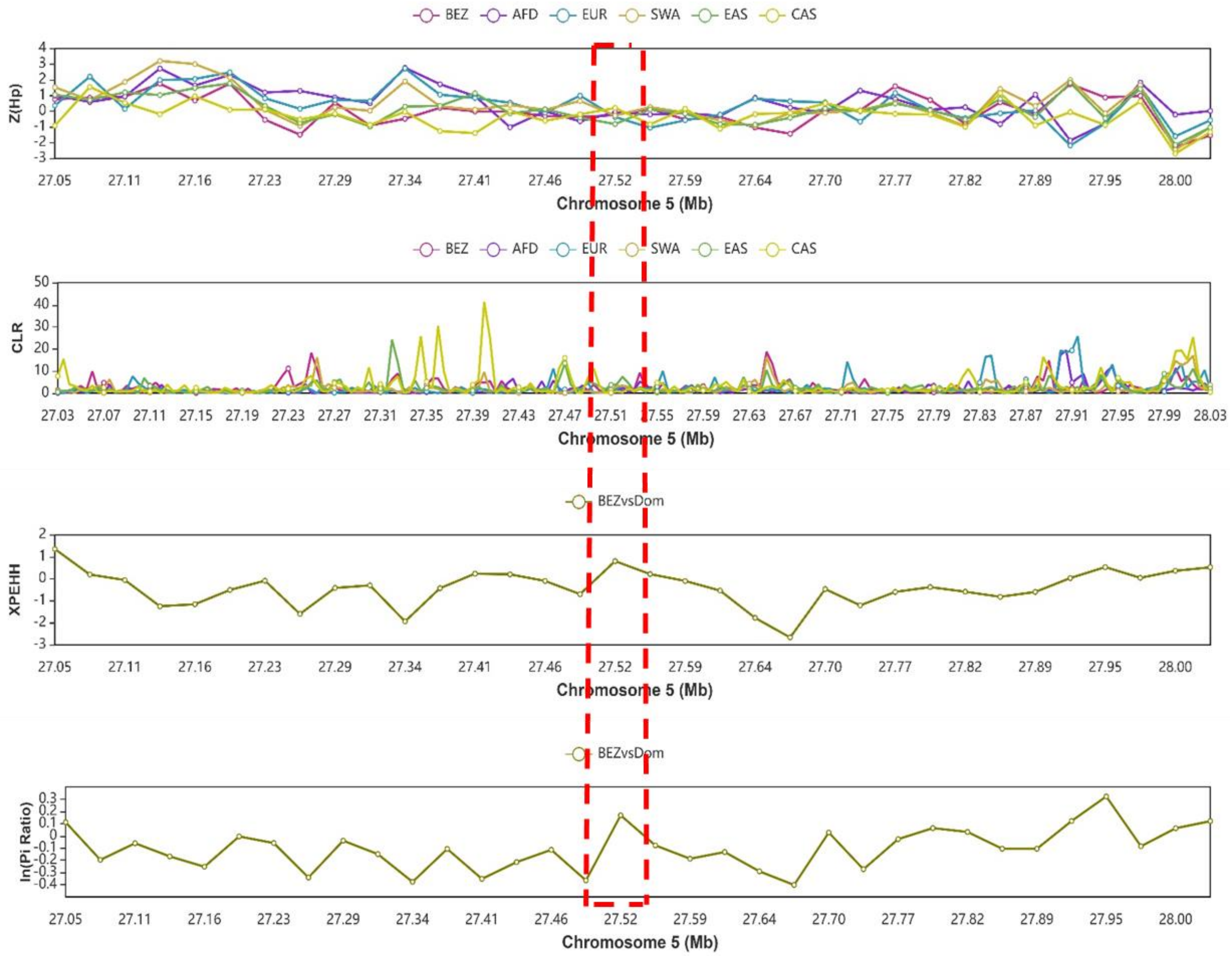

Figure S3. Selective sweep analysis across different statistical methods for the 0.5 Mb flanking region of the *NR4A1* gene: heterozygosity (Z(Hp)), composite likelihood ratio (CLR), cross-population extended haplotype homozygosity (XPEHH), and difference in nucleotide diversity (ln(Pi ratio)). The red dashed box referred to the genomic location of the *NR4A1* gene. Ancestral goat groups Bez = Bezoar, AFD = Africa dairy, EUR = Europe, SWA = Southwest Asia, EAS = East Asia, CAS = Cashmere, Dom = Domestic goat. Mb = megabyte
