# Supplementary material for: Functional and Genetic Insights into the Role of the NR4A1 Gene in the Litter Size of the Shaanbei White Cashmere Goat
Source: Animals (Basel). 2025 Jun 11;15(12):1729. doi: 10.3390/ani15121729 (PMC12189067; doi:10.3390/ani15121729)
Supplement: Supplementary file 1 [file animals-15-01729-s001.zip › Suplmentary File Table.pdf]

**Table S1.** Allele frequencies distribution of worldwide goat groups for the 11 bp-del locus of the *NR4A1* gene

| **Goat group** | ***N*** | **Reference Allele Frequencies** | **Alternate Allele Frequencies** |
|---|---|---|---|
| Bezoar | 24 | 0.771 | 0.229 |
| Morocco | 44 | 0.693 | 0.307 |
| Sudan | 6 | 0.75 | 0.25 |
| Nigeria | 2 | 0.75 | 0.25 |
| Ethiopia | 2 | 1 | 0 |
| Kenya, Toggenburg | 1 | 1 | 0 |
| Tanzania, Saanen | 7 | 0.571 | 0.429 |
| Tanzania, Toggenburg | 8 | 0.938 | 0.062 |
| Italy | 5 | 0.6 | 0.4 |
| Netherlands | 3 | 0.833 | 0.167 |
| Spain | 2 | 1 | 0 |
| Switzerland | 6 | 1 | 0 |
| Switzerland, Saanen | 9 | 0.722 | 0.278 |
| France | 6 | 0.833 | 0.167 |
| Australia | 4 | 1 | 0 |
| Iran | 36 | 0.736 | 0.264 |
| Barbari | 1 | 1 | 0 |
| Beetal | 5 | 1 | 0 |
| Bangladesh | 3 | 1 | 0 |
| Longlin | 6 | 1 | 0 |
| Leizhou | 6 | 1 | 0 |
| Southern China | 12 | 0.958 | 0.042 |
| Northern China | 16 | 0.875 | 0.125 |
| Cashmere | 9 | 0.611 | 0.389 |
| Tibet | 8 | 0.812 | 0.188 |

Where: *N* = number of genotyped animals

**Table S2.** Selected non-significantly associated resequencing loci SNPs of the *NR4A1* gene with goat litter size

| SNPs | Genotype | Genomic location | Variant ID | *N* | Mean ± SEM | *P*-Value |
|---|---|---|---|---|---|---|
| | TT | | | 44 | 1.73±0.09 | |
| SNP1 | TC | NC 030812.1: g. 27520274T>C | rs640733812 | 58 | 1.52±0.10 | 0.309 |
| | CC | | | 18 | 1.72±0.20 | |
| | GG | | | 71 | 1.61±0.08 | |
| SNP2 | GC | NC 030812.1: g. 27520294G>C | rs655140383 | 42 | 1.60±0.11 | 0.319 |
| | CC | | | 7 | 2.00±0.22 | |
| | GG | NC 030812.1: g. 27520821G>A | | 71 | 1.59±0.08 | |
| SNP3 | GA | | rs666131089 | 44 | 1.61±0.11 | 0.180 |
| | AA | | | 5 | 2.20±0.20 | |
| | TT | | | 46 | 1.72±0.09 | |
| SNP4 | TA | NC 030812.1: g. 27520982T>A | rs643733152 | 57 | 1.54±0.10 | 0.486 |
| | AA | | | 17 | 1.64±0.19 | |
| | TT | | | 15 | 1.73±0.15 | |
| SNP5 | TC | NC 030812.1: g. 27521294T>C | Novel | 46 | 1.50±0.11 | 0.313 |
| | CC | | | 59 | 1.69±0.09 | |
| | GG | | | 82 | 1.66±0.08 | |
| SNP6 | GT | NC 030812.1: g. 27521295G>T | Novel | 37 | 1.57±0.12 | 0.521 |
| | TT | | | 1 | 1.00 | |
| | AA | | | 77 | 1.62±0.07 | |
| SNP7 | AC | NC 030812.1: g. 27521298A>C | Novel | 38 | 1.66±0.14 | 0.748 |
| | CC | | | 5 | 1.40±0.24 | |
| | TT | | | 15 | 1.73±0.15 | |
| SNP8 | TC | NC 030812.1: g. 27521373T>C | rs646907046 | 46 | 1.50±0.11 | 0.313 |
| | CC | | | 59 | 1.69±0.09 | |
| | AA | | | 79 | 1.65±0.08 | |
| SNP9 | AG | NC 030812.1: g. 27521450A>G | rs672253455 | 38 | 1.63±0.12 | 0.305 |
| | GG | | | 3 | 1.00±0.00 | |
| | CC | | | 56 | 1.66±0.09 | |
| SNP10 | CT | NC 030812.1: g. 27521841C>T | rs660281089 | 51 | 1.61±0.11 | 0.836 |
| | TT | | | 13 | 1.54±0.24 | |
| | CC | | | 95 | 1.57±0.07 | |
| SNP11 | CT | NC 030812.1: g. 27521987C>T | rs648991538 | 23 | 1.78±0.13 | 0.192 |
| | TT | | | 2 | 2.50 | |
| | GG | | | 80 | 1.66±0.08 | |
| SNP12 | GA | NC 030812.1: g. 27521994G>A | rs659427647 | 39 | 1.56±0.11 | 0.481 |
| | AA | | | 1 | 1.00 | |
| | CC | NC 030812.1: g. 27522096T>C | | 46 | 1.72±0.09 | |
| SNP13 | CT | | Novel | 56 | 1.55±0.10 | 0.541 |
| | TT | | | 18 | 1.61±0.18 | |
| SNP14 | TT | NC 030812.1: g. 27522134T>C | Novel | 107 | 1.65±0.07 | 0.198 |
| | TC | | | 13 | 1.38±0.14 | |
| | CC | NC 030812.1: g. 27522240C>T | | 83 | 1.65±0.08 | |
| SNP15 | CT | | rs651977437 | 36 | 1.58±0.12 | 0.638 |
| | TT | | | 1 | 1.00 | |

| | | | | | | |
|---|---|---|---|---|---|---|
| SNP16 | GG<br>GA | NC 030812.1: g. 27524506G>A | rs685727717 | 102<br>18 | 1.58±0.07<br>1.89±0.18 | 0.088 |
| SNP17 | TT<br>TG | NC 030812.1: g. 27525788T>G | Novel | 117<br>3 | 1.62±0.07<br>2.00±0.58 | 0.357 |
| SNP18 | TT<br>TC<br>CC | NC 030812.1: g. 27527113T>C | rs664545959 | 57<br>71<br>12 | 1.68±0.09<br>1.57±0.10<br>1.58±0.26 | 0.688 |
| SNP19 | AA<br>AG | NC 030812.1: g. 27527808A>G | rs684908786 | 102<br>18 | 1.58±0.07<br>1.89±0.18 | 0.088 |
| SNP20 | TT<br>TC<br>CC | NC 030812.1: g. 27527910T>C | rs656174462 | 52<br>57<br>11 | 1.56±0.10<br>1.67±0.10<br>1.73±0.19 | 0.645 |
| SNP21 | CC<br>CT<br>TT | NC 030812.1: g. 27528220C>T | Novel | 81<br>19<br>17 | 1.56±0.08<br>1.74±0.15<br>1.94±0.16 | 0.104 |
| SNP22 | AA<br>AT<br>TT | NC 030812.1: g. 27528790A>T | Novel | 96<br>22<br>2 | 1.56±0.07<br>1.82±0.13<br>2.50 | 0.087 |
| SNP23 | CC<br>CT<br>TT | NC 030812.1: g. 27528885C>T | Novel | 58<br>50<br>12 | 1.64±0.08<br>1.62±0.11<br>1.58±0.26 | 0.970 |
| SNP24 | GG<br>GA | NC 030812.1: g. 27529672G>A | rs683570711 | 102<br>8 | 1.58±0.07<br>1.89±0.18 | 0.088 |
| SNP25 | CC<br>CA<br>AA | NC 030812.1: g. 27530103C>A | rs669599010 | 91<br>28<br>1 | 1.66±0.07<br>1.50±0.14<br>2.00 | 0.303 |
| SNP26 | CC<br>CG<br>GG | NC 030812.1: g. 27531351C>G | rs646413311 | 77<br>42<br>1 | 1.66±0.08<br>1.57±0.11<br>1.00 | 0.508 |
| SNP27 | GG<br>GC | NC 030812.1: g. 27531952G>C | rs639631459 | 102<br>18 | 1.58±0.07<br>1.89±0.18 | 0.088 |
| SNP28 | AA<br>AG<br>GG | NC 030812.1: g. 27532078A>G | rs639407986 | 1<br>22<br>97 | 1.00<br>1.68±0.12<br>1.62±0.08 | 0.708 |
| SNP29 | GG<br>GA<br>AA | NC 030812.1: g. 27532446G>A | rs659816764 | 77<br>42<br>1 | 1.66±0.08<br>1.57±0.11<br>1.00 | 0.508 |
| SNP30 | GG<br>GA | NC 030812.1: g. 27533622G>A | Novel | 25<br>9 | 1.56±0.13<br>2.00±0.24 | 0.365 |
| SNP31 | CC<br>CT | NC 030812.1: g. 27533683C>T | rs666019418 | 117<br>3 | 1.62±0.07<br>2.00±0.58 | 0.357 |
| SNP32 | TT<br>TG<br>GG | NC 030812.1: g. 27534006T>G | rs268259071 | 16<br>56<br>48 | 1.69±0.15<br>1.66±0.09<br>1.56±0.11 | 0.731 |
| SNP33 | AA<br>AG<br>GG | NC 030812.1: g. 27534901A>G | rs651878405 | 33<br>67<br>20 | 1.70±0.12<br>1.63±0.09<br>1.50±0.14 | 0.623 |

| | | | | | | |
|---|---|---|---|---|---|---|
| | CC | | | 82 | 1.59±0.07 | |
| SNP34 | CT | NC 030812.1: g. 27535171C>T | Novel | 33 | 1.73±0.16 | 0.797 |
| | TT | | | 5 | 1.60±0.24 | |
| | CC | | | 41 | 1.68±0.11 | |
| SNP35 | CT | NC 030812.1: g. 27536192C>T | rs675537086 | 53 | 1.58±0.09 | 0.784 |
| | TT | | | 24 | 1.67±0.17 | |
| | CC | | | 42 | 1.71±0.11 | |
| SNP36 | CT | NC 030812.1: g. 27536294C>T | rs652331080 | 53 | 1.62±0.11 | 0.521 |
| | TT | | | 22 | 1.50±0.13 | |

Where: SNP = Single nucleotide polymorphism; *N* = number of genotyped goats
